# Comparative transcriptomics of *Gymnosporangium* spp. teliospores reveals a conserved genetic program at this specific stage of the rust fungal life cycle

**DOI:** 10.1186/s12864-019-6099-x

**Published:** 2019-10-09

**Authors:** Si-Qi Tao, Bin Cao, Emmanuelle Morin, Ying-Mei Liang, Sébastien Duplessis

**Affiliations:** 1 The Key Laboratory for Silviculture and Conservation of Ministry of Education, Beijing Forestry University, Beijing, 100083 China; 2 State Key Laboratory of Mycology, Institute of Microbiology, Chinese Academy of Sciences, Beijing, 100101 China; 3 Université de Lorraine, Institut National de la Recherche Agronomique, Unité Mixte de Recherche 1136 Interactions Arbres-Microorganismes, Champenoux, France; 4 Museum of Beijing Forestry University, Beijing Forestry University, Beijing, 100083 China

**Keywords:** *Gymnosporangium*, Pucciniales, Secretome, Phylogenomics, Candidate effectors

## Abstract

**Background:**

*Gymnosporangium* spp. are fungal plant pathogens causing rust disease and most of them are known to infect two different host plants (heteroecious) with four spore stages (demicyclic). In the present study, we sequenced the transcriptome of *G. japonicum* teliospores on its host plant *Juniperus chinensis* and we performed comparison to the transcriptomes of *G. yamadae* and *G. asiaticum* at the same life stage, that happens in the same host but on different organs.

**Results:**

Functional annotation for the three *Gymnosporangium* species showed the expression of a conserved genetic program with the top abundant cellular categories corresponding to energy, translation and signal transduction processes, indicating that this life stage is particularly active. Moreover, the survey of predicted secretomes in the three *Gymnosporangium* transcriptomes revealed shared and specific genes encoding carbohydrate active enzymes and secreted proteins of unknown function that could represent candidate pathogenesis effectors. A transcript encoding a hemicellulase of the glycoside hydrolase 26 family, previously identified in other rust fungi, was particularly highly expressed suggesting a general role in rust fungi. The comparison between the transcriptomes of the three *Gymnosporangium* spp. and selected Pucciniales species in different taxonomical families allowed to identify lineage-specific protein families that may relate to the biology of teliospores in rust fungi. Among clustered gene families, 205, 200 and 152 proteins were specifically identified in *G. japonicum*, *G. yamadae* and *G. asiaticum*, respectively, including candidate effectors expressed in teliospores.

**Conclusions:**

This comprehensive comparative transcriptomics study of three *Gymnosporangium* spp. identified gene functions and metabolic pathways particularly expressed in teliospores, a stage of the life cycle that is mostly overlooked in rust fungi. Secreted protein encoding transcripts expressed in teliospores may reveal new candidate effectors related to pathogenesis. Although this spore stage is not involved in host plant infection but in the production of basidiospores infecting plants in the Amygdaloideae, we speculate that candidate effectors may be expressed as early as the teliospore stage for preparing further infection by basidiospores.

## Background

Asian juniper trunk rust disease caused by *Gymnosporangium japonicum* is a major threat to juniper (*Juniperus chinensis* L.) plantations throughout China as well as in the rest of Asia. *J. chinensis* is widely planted as a landscaping tree in parks, gardens and Chinese temples, also its wood of high density and decay resistance properties makes it broadly used in construction, furniture, and pulp industries [1]. However, the ornamental and economic significances have been severely affected by the rust disease. Formation of *G. japonicum* teliospores causes tree shape malformation and the trunk becomes gradually cracked [2, 3].

**Fig. 1:**
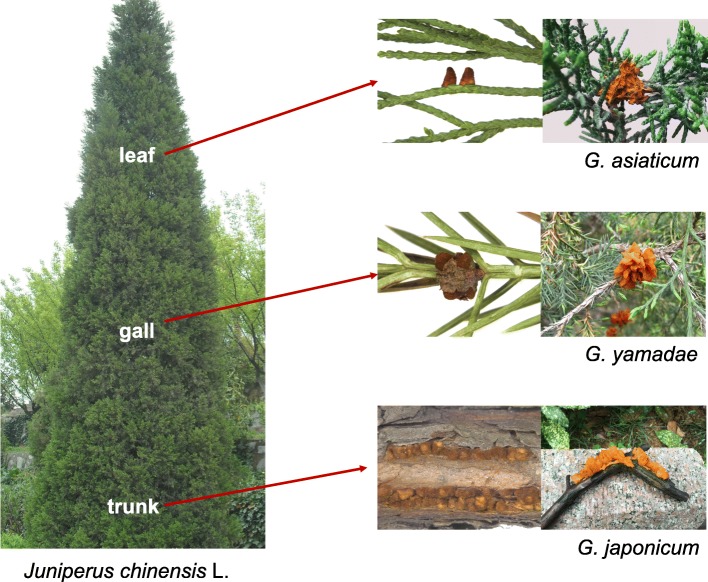
Illustration of rust disease symptoms caused by three *Gymnosporangium* species (*G. asiaticum*, *G. yamadae* and *G. japonicum*) on different organs of juniper (*J. chinensis* L.). Conical telia of *G. asiaticum* are folicolous and sometimes aggregated on witches’ broom; tongue-shaped telia of *G. yamadae* are formed on juniper gall, causing twigs dieback; whereas edge-shaped telia of *G. japonicum* are formed on trunk, causing tree shape malformation and gradually cracked trunk. Telia and teliospores can absorb water and become gelatinous with bright-yellow colour after a heavy rain

*G. japonicum* exhibits a typical complex rust life cycle with four spore types and two alternate hosts (*J. chinensis* for the telial stage and *Photinia villosa* for the aecial stage). Most *Gymnosporangium* are known to be heteroecious and demicyclic, and all reported telial hosts are gymnosperms, which is different from most rust fungi for which gymnosperms are aecial hosts [2, 4]. From phylogenetic studies of the Pucciniales, *Gymnosporangium* evidently appears as an undescribed family-level lineage rather than a sub-group of Pucciniaceae [5]. *G. japonicum*, *Gymnosporangium asiaticum* and *Gymnosporangium yamadae* are the three most widespread *Gymnosporangium* species in Asia and the two later species are the causal agents of Japanese apple rust and Japanese pear rust diseases, respectively [6]. They all share *J. chinensis* as a telial host, but the parasitic symptoms are formed on different organs of the tree (Fig. 1). Telia formation is a relatively well conserved feature within *Gymnosporangium* spp. [6], however, unlike the two other *Gymnosporangium* species commonly found on *J. chinensis*, *G. yamadae* and *G. asiaticum*, telia formed by *G. japonicum* are mainly found on hard tissues such as trunks and branches [2]. In spring, wedge-shaped telial horns full of teliospores extrude from the juniper trunk and arrange in rows. After a heavy rain, telia and teliospores become gelatinous with bright-yellow colour and generate airborne basidiospores which can transfer to the aecial host *P. villosa* [2]. Once a compatible interaction is established between the host and *G. japonicum*, pycnia emerge on the upper surface of the host leaves within a few days. After cross-fertilization of pycniospores, tubular aecia are formed after a few more days on the lower surface of infected leaves, which produce dikaryotic aeciospores that can infect the junipers again (see [4] or [7] for more details about the rust demicyclic life cycle). *G. japonicum* overwinters as mycelium under the bark of the trunk of juniper, and telia emerge the next year or several years later when environmental conditions are suitable [8, 9]. An ultrastructure analysis of *Gymnosporangium* telia revealed that the meiosis takes place in teliospores shortly after karyogamy and continues without interruption [10]. On the contrary, the meiosis process of rust species with overwintering telia can begin before winter and can remain in meiosis until teliospores germinate in the next spring [11, 12]. In spite of the economic importance and the biological singularity of *Gymnosporangium* spp., there is very limited knowledge about molecular processes controlling the life cycle of these fungi. More particularly, there is no molecular data available for *G. japonicum*.

The availability of genome resources has greatly supported advanced research on rust fungi by identifying conserved features shared by different Pucciniales lineages [13]. However, the number of sequenced genomes is still limited for rust fungi compared to other fungal groups, and the large genome size of rust fungi hinders rapid progress for some taxonomical families within the Pucciniales [5, 14]. Transcriptomics has granted additional support towards the basic understanding of the rust functional genome through the use of DNA-arrays and more recently RNA-seq, allowing the study of different life stages [15, 16]; the prediction of secretome and candidate pathogenesis factors [17–19]; and the survey of molecular interaction between rust fungi and their hosts (see review [13] for details). However, rust transcriptomics has mostly focused on urediniospores, particularly in macrocyclic rust fungi [7]. There is very limited information about other stages or about rust fungi that exhibits life cycles different than macrocyclic rust species [11, 15, 16, 20]. Only a few studies have focused on the telial stage, which is crucial in the life cycle of demicyclic species such as *Gymnosporangium* [11, 21, 22]. In the absence of a reference genome for *Gymnosporangium* spp., almost no molecular information is available for this genus. Recently, expression profiling of teliospores of *G. yamadae* and *G. asiaticum* was performed by RNA-seq [22]. The comparison of the two transcriptomes identified many highly expressed transcripts shared in the two species at this stage, as well as transcripts also represented in the genomes of reference rust species like *Melampsora larici-populina* or *Puccinia* spp. [22]. Such high-throughput transcriptome studies are invaluable to provide prospect to understand gene functions and metabolic pathways in this rust group.

In this study, we performed an RNA-seq analysis to characterize the transcriptome of *G. japonicum* at the telial stage (infection of *J. chinensis*) and we determined the functional annotation of this life cycle stage with an emphasis on the secretome. We also took advantage of previously published transcriptome data in two other *Gymnosporangium* species at the same life cycle stage to identify lineage-specific gene families compared to other rust fungi, including candidate secreted effector proteins and carbohydrate active enzymes (CAZymes).

## Results

### Transcriptome of *G. japonicum* teliospores

*G. japonicum* teliospores were collected from infected juniper branches in the Jiangsu province (China). Telia were visible in the form of a dense fungal mass of dark orange colour breaking through the bark of the juniper branch (Fig. 1). Teliospores were recovered by gently scratching the telia with a needle and inspection by light microscopy revealed typical two-cells spores attached to their pedicels and no contamination by plant material. Three biological replicates of *G. japonicum* teliospores were collected for total RNA extraction and were subjected to paired-end sequencing on Illumina Hiseq 2000 platform. In total, 49,245,136; 60,133,718; and 52,751,538 reads were obtained, respectively for the three replicates. After filtering low-quality reads and adapter sequences, we obtained a total of approximately 160 million clean reads (Additional file 1: Figure S1), which served as the input for an assembly with the Trinity program [23]. The clean reads of each replicate library were then mapped against this reference assembly and read count for each unigene was obtained using the RNA-Seq by Expectation Maximization (RSEM) software [24]. The mapping rates for each library were 82.9, 84.8 and 84.7%, respectively (Additional file 1: Figure S1). The generated assembly was composed of 40,583 transcripts, with a mean length of 1059-bp and 30,243 unigenes remained after elimination of transcripts redundancy (Additional file 1: Figure S1). A total of 15,722 CDS were determined from the unigenes using ESTscan and Blast against nr and swissprot as previously described [22]. Unigenes and transcript sequences are provided in the Additional files 2 and 3: Supplementary data 1 and 2. To further clarify the gene expression abundance in each library, FPKM values (fragments per Kb per million mapped reads) were calculated for each unigene (Additional file 4: Table S1). The FPKM density distribution profiles reflected consistent expression pattern across replicates (Additional file 5: Figure S2). Average FPKM value of the three replicates was further considered for all unigenes as a representative transcriptome of *G. japonicum* teliospores (Additional file 4: Table S1). In order to perform comparison across the *Gymnosporangium* lineage, we used transcriptome data previously published for *G. yamadae* and *G. asiaticum* teliospores [22]. The collection time (stage and time of the year in the life cycle) and the stage of maturation of teliospores on the same host tree, *J. chinensis*, were identical for the three *Gymnosporangium* species. The treatment of sequencing data and the assembly of unigenes performed in the transcriptome study by Tao and collaborators in 2017 [22] and in the present study were strictly identical. The Additional file 1: Figure S1 presents the sequencing metrics, and transcripts and unigenes assemblies which are all in very close range in term of numbers and unigenes length distribution. In some of the following sections, data from the three *Gymnosporangium* species were used for cross-comparison. In the *G. japonicum* teliospores transcriptome, 30,243 unigenes were identified, from which 15,722 CDS could be defined. These data served as a basis for annotation and comparison to transcriptomes from other *Gymnosporangium* spp.

### Functional overview of genes expressed in *G. japonicum* teliospores

**Fig. 2:**
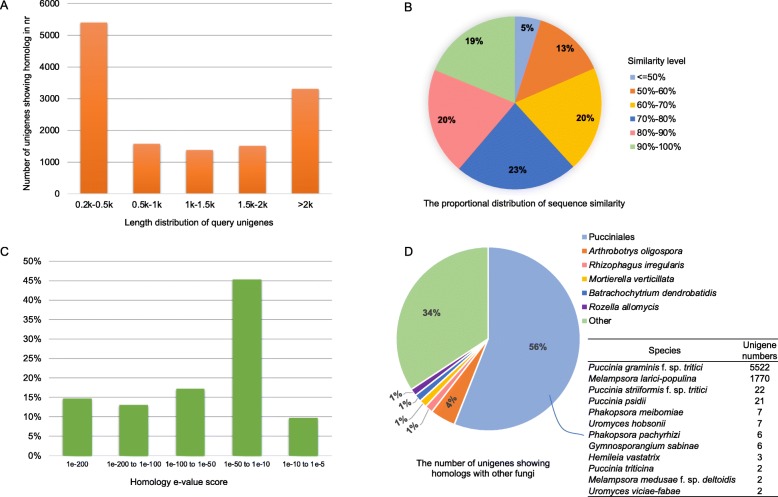
Homology of *Gymnosporangium japonicum* unigenes in the NCBI non-redundant (nr) database (blastx, e-value ≤10^−5^). **a**: Length distribution of *G. japonicum* unigenes with homologs in nr. **b**: Proportional distribution of similarity levels of *G. japonicum* unigenes with homologs in nr. **c**. E-value distribution of *G. japonicum* unigenes with homologs in nr. **d**: Proportional distribution by species of *G. japonicum* unigenes with homology in nr (details in the rust fungal order Pucciniales are provided)

To obtain a detailed description and a functional annotation of the *G. japonicum* teliospores transcriptome, six public databases including the NCBI non-redundant (nr) and nucleotide (nt) databases, protein family (Pfam), SwissProt, eukaryotic orthologous groups (KOG), gene ontology (GO), and Kyoto encyclopaedia of genes and genomes (KEGG) databases were selected for similarity searches using Basic Local Alignment Search Tools (blastx and tblastx; e-value <1e^− 5^). A total of 18,872 unigenes showed similarity to at least one database and 2405 unigenes were annotated in all selected databases. The Table 1 gives the number of unigenes showing similarity in the seven databases and the details for each unigene can be retrieved in the (Additional file 4: Table S1). The nr database showed the largest number of homologs (13,165; 43.5%) for the *G. japonicum* unigenes set, which is detailed hereafter. Among these 13,165 unigenes, 41 and 25.1% had a length ranging between 200 and 500 bp, or larger than 2 Kbp, respectively (Fig. 2a). Besides, 39 and 56% of the unigenes showed similarity levels higher than 80%, and between 50 and 80%, respectively (Fig. 2b). Although about 45% of the 13,165 unigenes showed high homology e-values (ranging between 1e-50 to 1e-200), 45.2% of the unigenes with homology in nr had an e-value ranging between 1e-50 and 1e-10 (Fig. 2c). More than half of the 13,165 unigenes (56%) showed homology to Pucciniales sequences, notably 5521 (41.9%) and 1770 (13.4%) unigenes matched with *Puccinia graminis* f. sp. *tritici* and *M. larici-populina*, respectively (Fig. 2d). We also determined whether transposons were abundant in the dataset by comparing unigenes to Repbase (https://girinst.org/repbase/; blastx, e-value ≤10–5) and searching for transposon-related annotations in the results of the similarity search against nr. Only 75 and 138 unigenes presented similarity to repbase and transposon-related sequences in nr, respectively, indicating that transposons were marginal in the dataset.

**Table 1:** Annotation of *Gymnosporangium japonicum* unigenes in selected public databases

	Number of Unigenes	Percentage (%)
Annotated in NR	13,165	43.5
Annotated in NT	8421	27.8
Annotated in KO (KEGG Ortholog)	5444	18
Annotated in SwissProt	11,510	38
Annotated in PFAM	11,624	38.4
Annotated in GO	12,309	40.7
Annotated in KOG	7937	26.2
Annotated in all Databases	2405	8
Annotated in at least one Database	18,872	62.4
Total Unigenes	30,243	100

Homology with KOG and KEGG databases were scrutinized to determine gene functions and metabolic pathways expressed in *G. japonicum* teliospores (Additional file 6: Table S2). A total of 7937 unigenes (26.3% of all unigenes) were assigned to 25 KOG functional categories. Unigenes falling in the “posttranslational modification, protein turnover, chaperones” category constituted the largest proportion (3.7%), followed by the category “translation, ribosomal structure and biogenesis” (3.4%). After genes placed in the category “general function prediction only”, a total of 2 and 1.9% of the expressed genes were classified in the “energy production and conversion” and “signal transduction mechanisms” categories, respectively. Of the 5444 unigenes assigned to 32 KEGG pathways, the three most important pathways were “translation” (913 unigenes), “carbohydrate metabolism” (669 unigenes) and “signal transduction” (605 unigenes) (Additional file 6: Table S2).

The distribution of the mean expression levels of unigenes across the *G. japonicum* teliospores replicates revealed a large proportion of unigenes with a low expression value (e.g. 12,480 unigenes with a Log_10_FPKM value below 0; Additional file 7: Figure S3), which is similar to observations made in transcriptomes of other *Gymnosporangium* spp. [22]. On the other hand, 17,763 and 6511 unigenes showed a Log_10_FPKM value above 0 and 1, respectively. A total of 977 unigenes showed a high expression level above 2 Log_10_FPKM, among which 65 showed a very high expression level above 3 Log_10_FPKM (Additional file 7: Figure S3). Among these, 44 (68%) unigenes had a homolog in the nr database (including 25 and 15 with the rust fungi *P. graminis* f. sp. *tritici* and *M. larici-populina*, respectively), and more than half of these unigenes encode hypothetical proteins with unknown function (Table 2). Two histones (GYJ|c28242_g1, GYJ|c12350_g1) and one glycoside hydrolase 26 (GH26, GYJ|c8249_g1) were among the most highly expressed unigenes. We searched all unigenes that belonged to teliospore-associated KOG categories based on previous transcriptomes for rust fungal teliospores [11, 21]. We found 26 unigenes encoding aquaporins, 3 unigenes encoding calcium transporting ATPase, 24 unigenes coding for pleiotropic drug resistance and 17 unigenes encoding multicopper oxidase laccase like proteins expressed in *G. japonicum* teliospores. Also, a large number of meiosis-related genes were found at this stage, including Dmc1, Spo11, Rec8 encoding meiotic recombination proteins; Mre1, Rad50, Rad51 coding for double-strand break repair proteins; the meiotic nuclear division protein Mnd1 and the meiotic cell division protein Dom34; and the meiotic checkpoint regulator Tsg24 (Additional file 4: Table S1). A total of 18,872 *G. japonicum* unigenes from the teliospores transcriptome were annotated according to diverse databases, allowing to derive subsets of highly expressed unigenes in functional categories.

**Table 2:** 65 most highly expressed unigenes (FPKM value > 1000) in *G. japonicum* teliospores

Transcript_ID^a^	FPKM^b^	Length^c^	SP^d^	Description^e^	Blast gene_ID
c6032_g1	107,637.9	325	no	No hit found	–
c28236_g2	56,894.52	850	no	Hypothetical protein	TREMEDRAFT_42124
c28242_g1	18,140.33	950	no	Histone	PGTG_00393
c7790_g2	17,703.31	1449	yes	Hypothetical protein	PGTG_17690
c10708_g1	14,201.34	1786	no	No hit found	–
c11262_g1	13,148.50	1085	no	Secreted protein	MELLADRAFT_95585
c25177_g1	12,430.72	689	no	Hypothetical protein	MELLADRAFT_88098
c11902_g1	11,733.81	2231	no	Hypothetical protein	G7K_1408-t1
c9515_g1	11,376.58	1658	yes	Hypothetical protein	PGTG_17079
c11318_g1	10,971.72	1393	no	Hypothetical protein	PGTG_17690
c22047_g1	9192.39	928	no	Hypothetical protein	PGTG_17334
c14339_g2	9139.16	1015	yes	Hypothetical protein	MELLADRAFT_35323
c2022_g1	8673.68	1414	no	No hit found	–
c25134_g2	7436.62	2599	yes	Subtilisin protease	MELLADRAFT_88167
c8249_g1	6750.63	1717	yes	Glycoside hydrolase 26	MELLADRAFT_106238
c14040_g1	6614.23	1244	yes	No hit found	–
c11851_g2	5537.22	1084	no	hypothetical protein	PGTG_19738
c14339_g1	5115.31	502	yes	Hypothetical protein	MELLADRAFT_35323
c2906_g1	4708.21	1667	yes	Hypothetical protein	MELLADRAFT_70656
c9022_g1	4088.89	1023	no	No hit found	–
c12099_g1	3900.42	2266	no	Hypothetical protein	PGTG_14475
c11851_g1	3684.44	272	no	No hit found	–
c16092_g2	3622.42	1607	no	No hit found	–
c10950_g1	3514.04	1732	yes	Secreted protein	MELLADRAFT_73055
c4527_g1	3051.66	4137	no	RAN protein kinase	PGTG_21658
c11948_g1	2965.60	414	no	No hit found	–
c11347_g1	2690.92	2048	yes	Hypothetical protein	PGTG_02189
c10791_g1	2632.66	1700	no	Hypothetical protein	PGTG_14864
c8994_g1	2612.53	3228	no	Hypothetical protein	PGTG_18470
c16092_g3	2604.10	1283	no	No hit found	–
c1786_g1	2533.90	1481	no	Hypothetical protein	MELLADRAFT_72697
c19079_g1	2505.70	1036	no	No hit found	–
c65_g1	2451.47	2298	yes	Hypothetical protein	MELLADRAFT_71192
c18890_g1	2414.35	619	no	Hypothetical protein	MELLADRAFT_88098
c15544_g1	2363.95	1282	no	Hypothetical protein	PGTG_03708
c10611_g1	2345.25	1689	yes	Secreted protein	MELLADRAFT_116225
c18913_g1	2331.34	2594	yes	Hypothetical protein	PGTG_10789
c4594_g1	2182.52	1616	yes	Hypothetical protein	PGTG_00638
c9046_g1	2023.74	2473	yes	Hypothetical protein	MELLADRAFT_73714
c13940_g1	1973.04	226	no	No hit found	–
c1034_g1	1968.25	465	no	No hit found	–
c25416_g1	1954.22	1363	no	No hit found	–
c7866_g1	1908.49	1777	no	Hypothetical protein	PGTG_07040
c28275_g1	1837.78	1927	no	No hit found	–
c12457_g1	1823.57	1381	no	No hit found	–
c7873_g1	1793.38	1991	no	Hypothetical protein	MELLADRAFT_49061
c14029_g2	1772.19	2196	no	Hypothetical protein	MELLADRAFT_72270
c12350_g1	1604.07	434	no	Histone	PGTG_01301
c7823_g1	1589.52	1667	no	Hypothetical protein	PGTG_11071
c31399_g1	1589.15	534	no	No hit found	–
c2267_g1	1524.26	852	yes	No hit found	–
c13642_g1	1452.79	2299	no	Hypothetical protein	PGTG_15958
c1160_g1	1438.03	3746	no	Hypothetical protein	PGTG_11520
c28418_g1	1422.03	997	no	Hypothetical protein	SPOSA6832_01986
c11513_g1	1290.63	1788	no	No hit found	–
c1787_g1	1232.38	2535	yes	Hypothetical protein	PGTG_16765
c9470_g1	1195.03	1451	no	No hit found	–
c25232_g1	1153.14	841	yes	No hit found	–
c16423_g1	1141.52	2216	no	Hypothetical protein	PGTG_02943
c10746_g1	1138.26	3450	no	Hypothetical protein	HMPREF1120_11008
c2397_g1	1115.61	2748	no	Hypothetical protein	PGTG_02069
c14952_g1	1090.41	4366	no	Hypothetical protein	PGTG_17785
c14366_g1	1050.03	3670	no	Cyclin B	PGTG_13538
c6040_g1	1043.71	2004	yes	Hypothetical protein	PGTG_00770
c10606_g1	1014.47	1166	no	No hit found	–

^a^ *Identity of transcript assembled from G. japonicum teliospores RNA-seq data*

^b^ *Average fragments per kb of exon model per million reads mapped value of unigenes from each cDNA library*

^c^ *Unigene length in nucleotides*

^d^ *Predicted secreted proteins*

^*e*^ *Functional annotation according to homology in the NCBI non-redundant database (best blastp hit)*

### Comparison of *Gymnosporangium* teliospore transcriptomes highlights commonly and specifically expressed genes

**Fig. 3:**
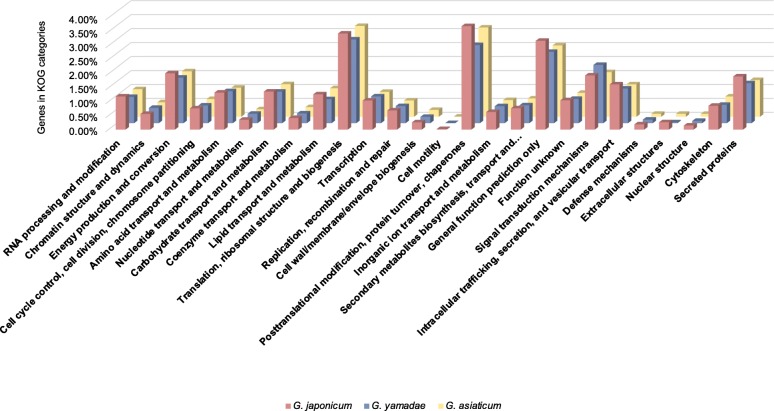
Distribution of unigenes from *G. japonicum*; and from *G. asiaticum* and *G. yamadae* in eukaryotic orthologous group (KOG) categories (x-axis). The main category corresponding to unknown function was removed for legibility. The manually annotated “secreted proteins” category refers to unigenes encoding predicted secreted proteins of unknown functions. Unigenes and proteomes of *G. yamadae* and *G. asiaticum* were retrieved from [22]

To get a better understanding of the functions expressed in the transcriptomes of *Gymnosporangium* spp. teliospores, we compared the transcriptomes of *G. japonicum*, *G. yamadae* and *G. asiaticum* teliospores which correspond to the same life cycle stage set on a same tree, *J. chinensis*, but in different organs [2, 22]. Distributions of unigenes in KOG categories were very similar in the surveyed *Gymnosporangium* spore stage (Fig. 3). In the three species, the number of unigenes without KOG functional annotation was predominant. Then, “posttranslational modification, protein turnover, chaperones” and “translation, ribosomal structure and biogenesis” categories were the most important annotated categories in the three species, followed by the categories “general function prediction only”; “energy production and conversion”; and “signal transduction mechanisms”. Since, *G. japonicum* teliospores are formed on woody tissues of the host tree *J. chinensis*, we gave a particular attention to CAZymes annotation, which are important enzymes involved in the plant cell wall decomposition [25]. For *G. japonicum*, *G. yamadae* and *G. asiaticum*, a total of 281, 284 and 297 unigenes were assigned to CAZymes families in the dbCAN database, respectively (Additional file 8: Table S3 and Additional file 9: Figure S4). Most CAZymes belonged to glycosyl hydrolases (GHs) families and only a few were grouped into carbohydrate binding modules families. Overall, the composition in each CAZyme type was relatively similar in *G. japonicum* compared with the two others (Additional file 9: Figure S4). Several unigenes belonging to the GH26 family, one from *G. japonicum* (GYJ|c4903_g1), two from *G. yamadae* (GYY|c14266_g2; GYY|c13067_g1) and four from *G. asiaticum* (GYA|c34482_g1; GYA|c1703_g1; GYA|c2116_g1; GYA|c20188_g1), were retrieved and in particular, one unigene from each species (GYJ|c4903_g1; GYY|c13067_g1 and GYA|c20188_g1) showed a higher expression level in teliospores. Other GHs from families 16, 17 and 18 showed high expression levels in the three *Gymnosporangium* spp. (Log_10_FPKM > 2; Additional file 8: Table S3), with different expression levels noticeable between members of these GH families in these three rust species. This result suggests that *Gymnosporangium* spp. may possess a common set of enzymes for plant cell wall decomposition and that expression reflects specific activities in infected host tissues. Overall, comparison of annotations of *G. japonicum*, *G. yamadae* and *G. asiaticum* unigenes indicates that the most highly represented functional KOG categories were similar in teliospores transcriptomes of the three rust species. Moreover, unigenes encoding CAZymes were similarly represented between *Gymnosporangium* spp. with particularly high expression profiles for specific GHs.

### Comparison of *Gymnosporangium* teliospores predicted proteomes and secretomes identifies commonly and specifically expressed secreted proteins

**Fig. 4:**
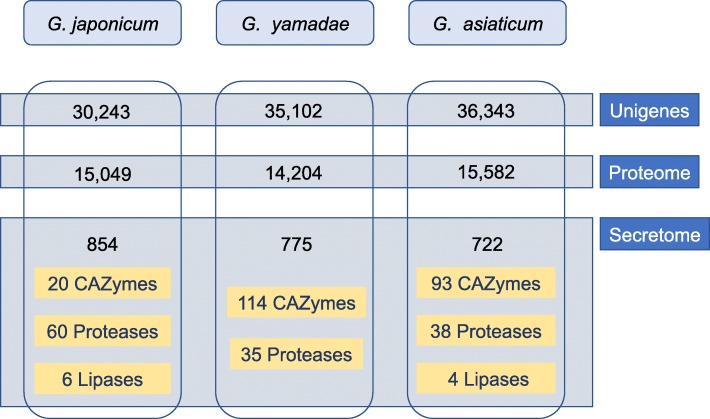
Proteomes and secretomes predicted from *G. japonicum*, *G. yamadae* and *G. asiaticum* unigenes. Proteomes were derived from unigenes using TransDecoder. Secretomes were predicted from proteomes using a combination of secretion predictors. Functional annotations of predicted secreted proteins were performed using the dbCAN, the Merops and the Lipase Engineering databases to identify putative carbohydrate active enzymes (CAZymes), proteases and lipases in each *Gymnosporangium* secretome. Unigenes and proteomes of *G. yamadae* and *G. asiaticum* were retrieved from [22]

**Fig. 5:**
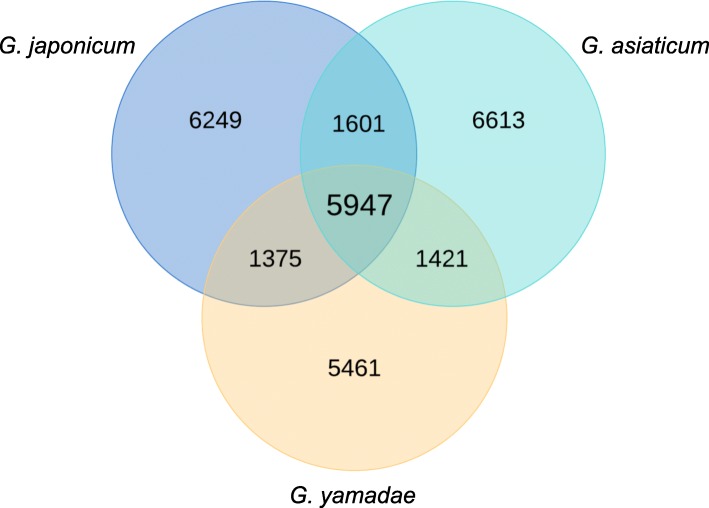
Conserved and species-specific proteins identified between *Gymnosporangium* spp. Proteins predicted from *G. japonicum* (this study) and *G. yamadae* and *G. asiaticum* [22] unigenes were compared and homologs were identified by searching reciprocal blast hits (Blastp; e-value ≤1e-5)

CDS were determined in *G. japonicum* unigenes based on similarity in databases, which cannot identify new specific proteins. TransDecoder predicted 15,049 proteins from *G. japonicum* unigenes (Fig. 4). In total, 10,917 of these predicted proteins showed similarity in the nr database, which is similar to the number observed with CDS (Table 1). Predicted proteomes from *G. asiaticum* and *G. yamadae* were retrieved from [22] and compared by reciprocal best hits (BLASTp; e-value ≤1e-5). We found 5947 proteins conserved in all three *Gymnosporangium* species (Fig. 5; Additional file 10: Table S4). Moreover, 7548 and 7322 orthologous proteins were found between *G. japonicum* and *G. asiaticum*; and *G. japonicum* and *G. yamadae*, respectively. There were 6249; 6613 and 5461 proteins specifically detected in *G. japonicum*, *G. asiaticum* and *G. yamadae*, respectively (Fig. 5; Additional file 10: Table S4).

**Fig. 6:**
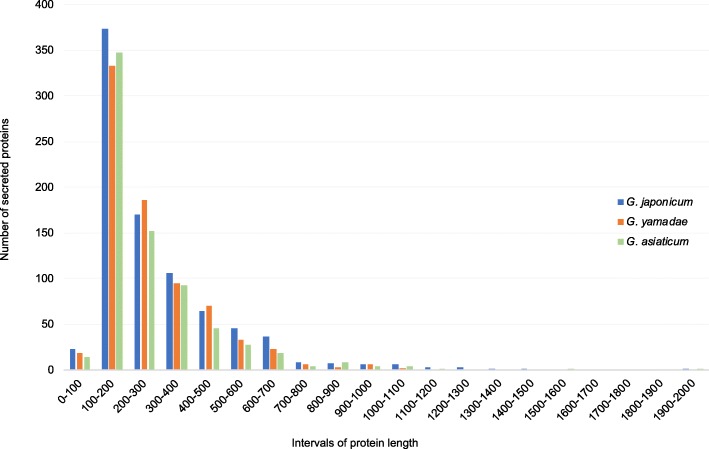
Length distribution of secreted proteins in the predicted secretomes of *G. japonicum*, *G. yamadae* and *G. asiaticum*

Rust fungi possess large secretomes, which contain candidate virulence effectors that play a key role in fungal pathogenicity [7]. These effectors are often small secreted proteins of unknown function. Secreted proteins (SPs) were identified in the transcriptome of *G. japonicum* using a dedicated pipeline of secretion predictors as previously described [26]. Among the 15,049 predicted proteins, 854 (5.67%) corresponded to SPs (Additional file 11: Table S5). The predicted SPs had a size varying between 99 and 1930 amino acids, with a median length of 215aa (Fig. 6). Within SPs, four are homologues of the conserved rust transferred protein RTP1 [27]. Typical secreted proteins of CAZyme, protease and lipase categories were searched in dedicated databases. In summary, 20 SPs belonged to 18 CAZymes families, 60 SPs were annotated in 24 proteases families and six SPs were part of two lipases families (Fig. 4). Among the top 65 highly expressed unigenes, 19 corresponded to predicted SPs and 16 showed similarity to *M. larici-populina* or *P. graminis* f. sp. *tritici* proteins, including a glycoside hydrolase and a protease (Table 2). The same secretome prediction pipeline was applied to the previously published transcriptomes of *G. yamadae* and *G. asiaticum* teliospores [22] to allow comparison within *Gymnosporangium* species (Additional file 11: Table S5). In total, 775 SPs were predicted for *G. yamadae*, with a size ranging from 99 to 1083 amino acids and a median size of 215. In *G. asiaticum*, 722 SPs were predicted, with a size ranging from 99 to 1981 amino acids, with a median length of 200 (Fig. 4 and Fig. 6; Additional file 11: Table S5). The size distribution for the secreted proteins of the three *Gymnosporangium* species is relatively uniform and small SPs account for the largest proportion (Fig. 6). Some SPs were annotated as CAZymes (114 for *G. yamadae* and 93 for *G. asiaticum*), proteases (35 for *G. yamadae* and 38 for *G. asiaticum*) and lipases (not found in *G. yamadae* and 4 for *G. asiaticum*) using dedicated databases (Fig. 4).

Based on the secretome prediction, unigenes of unknown function corresponding to SPs were classified in a manually annotated category termed “secreted protein” determined after [16]. A total of 577 *G. japonicum* unigenes were classified in this “secreted protein” KOG category, whereas, 480 and 504 *G. asiaticum* and *G. yamadae* unigenes were classified in the same KOG category, respectively. In the three *Gymnosporangium* spp., the “secreted protein” category is among the most abundant KOG categories after signal transduction, representing 1.9, 1.4 and 1.3% of the respective unigenes (Fig. 3).

To conclude, proteome and secretome predictions in the three *Gymnosporangium* teliospore transcriptomes identified hundreds of SPs of unknown functions that may represent putative candidate virulence effectors in each rust species. More particularly, many SPs are among highly expressed transcripts in teliospores and show similarity to other rust fungi. Interestingly, SPs of unknown function are similarly relatively abundant in the three *Gymnosporangium* spp.

### Comparison of *Gymnosporangium* spp. transcriptomes with Pucciniales genomes supports taxonomic proximity to the Pucciniaceae

**Fig. 7:**
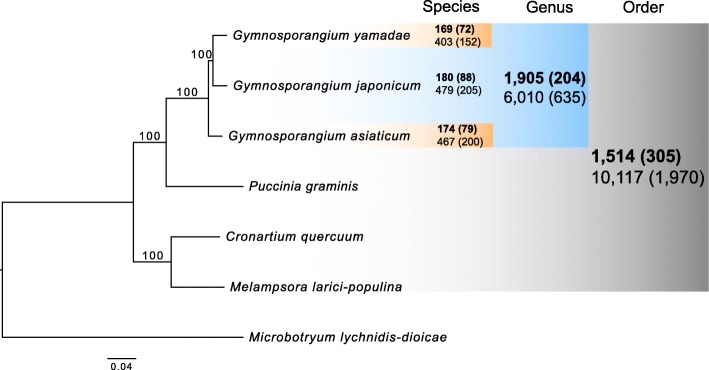
Phylogenomic relationship of three *Gymnosporangium* spp. with selected species in the Pucciniomycotina and identification of conserved proteins based on Multiclustering (MCL) analysis. A Maximum likelihood tree was constructed with RAxML using concatenated protein sequences from 1042 conserved single-copy genes found in the predicted proteomes of *Gymnosporangium* spp. teliospore transcriptomes and of four fungi selected in the Pucciniomycotina (*Puccinia graminis* f. sp. *tritici*, *Melampsora larici-populina*, *Cronartium quercuum* f. sp. *fusiforme* and *Microbotryum lychnidis-dioicae*). Bootstraps values are shown on branches (1000 replicates). Bold numbers shown at the species, genus or order levels, indicate lineage-specific clusters identified by the MCL analysis between the proteomes from the selected species, and bold numbers in bracket indicate the number of clusters remaining as specific after similarity search in the NCBI non-redundant protein database (Blastp; e-value ≤1e-5). The total number of proteins found in corresponding MCL clusters at each taxonomical level are shown non-bold below the cluster numbers

In order to identify lineage-specific proteins in *Gymnosporangium* spp. and beyond within the Pucciniales, we compared the three available *Gymnosporangium* transcriptomes with deduced proteomes of selected basidiomycetes. Three other rust fungi were selected as representatives of different taxonomical families within the order Pucciniales for which a reference genome was available (i.e. the Cronartiaceae, the Melampsoraceae, and the Pucciniaceae) [28–30]. *Microbotryum lychnidis-dioicae* was selected as an outgroup fungal pathogen in the Microbotryomycetes, a sister class of Pucciniomycetes within the Pucciniomycotina [31]. A total of 1042 single copy genes conserved across the selected Pucciniales genomes, the three *Gymnosporangium* spp. transcriptomes and the outgroup genome in Microbotryomycetes were predicted by BUSCO [32] and were used to build a maximum likelihood tree with RAxML [33] (Fig. 7). Two rust clades were evident: *C. quercuum* f. sp. *fusiforme* clustered with *M. larici-populina* whereas the *Gymnosporangium* species were grouped with *Puccinia graminis* f. sp. *tritici*; *G. japonicum* branching more closely to *G. yamadae* than to *G. asiaticum*.

### Comparison of *Gymnosporangium* spp. transcriptomes with Pucciniales identifies new specific rust fungal transcripts and putative candidate effectors

FastOrtho was used to conduct a Markov Cluster Algorithm (MCL) analysis of predicted proteomes of the seven selected fungal species [34]. Then, the specificity of proteins identified at any lineage level was assessed by comparison to the NCBI nr database (blastp, e-value ≤1e-5). A total of 1514 clusters (10,117 proteins) were identified for the six Pucciniales included in this study (Fig. 7; Additional file 12: Table S6). After filtering against the nr database, 1970 proteins within 305 clusters were considered as Pucciniales-specific (Fig. 7; Additional file 12: Table S6). To pinpoint protein families specific to the genus *Gymnosporangium*, we selected 1905 clusters, representing a total of 6010 proteins, which were only present in the three *Gymnosporangium* species after the MCL analysis (Fig. 7; Additional file 12: Table S6). After filtering against the nr database, 204 clusters representing a total of 635 proteins, were deemed as specific of *Gymnosporangium* spp. and specifically expressed in the life cycle stage teliospores (Fig. 7; Additional file 12: Table S6). Finally, at the species level, we found 180, 169 and 174 clusters (corresponding to 479, 403 and 467 proteins, respectively) that were specific to *G. japonicum*, *G. yamadae* and *G. asiaticum*, respectively (Fig. 7; Additional file 12: Table S6). After comparison to the nr database, 205 orphan proteins were specific to *G. japonicum*, of which 14 were predicted to encode SP, representing putative specific candidate effectors for this species. Additionally, there were 152 and 200 orphan proteins found in *G. yamadae* and *G. asiaticum*, respectively (Fig. 7; Additional file 12: Table S6). A total of 11 and 12 predicted secreted proteins were found in each of the later species, indicating candidate secreted effector proteins in *G. yamadae* and *G. asiaticum*, respectively. Previous genomic studies have revealed the presence of large secreted protein gene families in rust fungi that may represent diversifying candidate effector families [7, 35]. To identify such families within the three *Gymnosporangium* spp. transcriptomes, we selected clusters containing one or more SPs in each species (Additional file 13: Table S7). Among clusters with SPs, specific protein families containing only 3 to 4 SP members were retrieved (Additional file 13: Table S7). To conclude, the comparison of the teliospore transcriptomes with reference rust fungal genomes identified hundreds of orphans in each *Gymnosporangium* spp., as well as core proteins shared at the genus level or at the Pucciniales order, which informs us about the involvement of specific rust proteins at this stage of the life cycle.

## Discussion

In this study, we report the transcriptome profiling of *G. japonicum* teliospores and take advantage of the previously published transcriptomes of *G. yamadae* and *G. asiaticum* teliospores to perform comparison within this genus [22]. *G. japonicum*, *G. yamadae*, and *G. asiaticum* are the three most widespread *Gymnosporangium* species in Asian areas [6, 36]. They share *J. chinensis* as their telial host but they cause rust symptoms on different organs of juniper trees, which indicate major differences in their infection modes [2, 6]. In order to also unravel specificities of this life stage compared to heteroecious and macrocyclic rust fungi, we compare the three *Gymnosporangium* transcriptomes with predicted genes in the genomes of *C. quercuum* f. sp. *fusiforme*, *M. larici-populina* and *P. graminis* f. sp. *tritici* [28–30]. *Gymnosporangium* spp. may exhibit different gene expression patterns and metabolic processes at the teliospore stage on their unique telial host (gymnosperm) than heteroecious rust species [2, 4]. So far, only a few transcriptomic studies looked at gene expression at the telial stage in a few species [11, 21, 22]. Teliospores of macrocyclic rust fungi act as survival structures throughout winter on the telial host [7]. At the contrary, teliospores of *Gymnosporangium* species are not overwintering structures, they are produced transiently in spring under favourable conditions and they lead to the production of basidia then basidiospores within a few days [2, 37]. Basidiospores of *Gymnosporangium* have the capacity to infect plants of the subfamily Amygdaloideae, on which important damages can occur. It is crucial to understand the biology of this transient and unique life stage that differ from the corresponding one in macrocyclic rust fungi. So far, our understanding of the molecular processes at play in *Gymnosporangium* teliospores is still limited.

Rust fungi possess large numbers of genes and a majority have no known function, illustrating the need to better describe rust biology through global approaches such as transcriptomics [5]. This is illustrated by the low proportion of CDS predicted from the unigenes by similarity in nr and swissprot. A large proportion of highly expressed *Gymnosporangium* genes are annotated as proteins of unknown function with no homology in databases, indicating that teliospores undergo largely unknown biological processes. The distribution of unigenes annotated in KOG categories in the three *Gymnosporangium* teliospores transcriptomes suggests the expression of a conserved genetic program, despite the fact that each *Gymnosporangium* infect a different organ of *J. chinensis*. The most abundant cellular categories corresponded to translation, post-translation, signal transduction and energy production which supports that *Gymnosporangium* teliospores are highly active in spring, likely to prepare for the production of basidiospores and later infection of the aecial host. Plant pathogens like rust fungi secrete effector molecules inside cells of their host plants to manipulate immunity and physiology [38]. Secretome prediction allow to identify SPs that are candidate effectors that may play a role in pathogenesis [39–41]. Unigenes encoding predicted SP of unknown function likely contain candidate rust fungal effectors [41, 42]. With a total of 513 to 854 SPs detected in the three *Gymnosporangium* species, this category is also one of the most represented in expressed unigenes. Teliospores are not infecting living host tissues, thus the presence of a large number of expressed SPs maybe puzzling. However, teliospores lead to the production of basidia and basidiospores within only a few days in favourable conditions [2, 37] and it is tempting to speculate that candidate effector transcripts may be already produced at the teliospore stage to facilitate rapid infection of the aecial host by basidiospores. Also, it cannot be ruled out that secreted proteins may have unknown roles unrelated to host infection.

Functional annotation of top highly expressed genes in *G. japonicum* revealed a hemicellulose cleaving enzyme of the GH26 family and members of the GH16, 17, 18 families. Previous studies showed that the GH26 gene family is expanded in the genomes of *M. larici-populina* and *P. graminis* f. sp. *tritici* [28]. Unigenes presenting homology to GH26 genes were found in the three *Gymnosporangium* species with high expression levels. GH26 genes were previously shown to be highly expressed as well in *M. larici-populina* telia, urediniospores and during poplar infection [11, 28, 43]. Similarly, genes falling in this GH family were significantly induced in early needle infection stage of *C. ribicola* [15] and highly expressed in the urediniospores of *P. triticina* [29]. GH26 is a widespread hemicellulose-cleaving enzymes family conserved in rust species that may play an essential role in modifying plant cell wall to facilitate fungal invasion. Other functions previously reported in *M. larici-populina* and *P. triticina* teliospores, like multicopper oxidase encoding genes are represented among highly expressed genes of *Gymnosporangium* teliospores [11, 21]. Although the exact role of multicopper oxidase has yet to be resolved, our study completes the view of the functional transcriptome of teliospores and shows that this function might be critical at this stage. Finally, the composition in the different CAzyme categories was rather similar between the three *Gymnosporangium* species, indicating that the capacity to infect different organs of *J. chinensis* may not be reflected in the fungal equipment for plant cell wall decomposition, but rather in the expression or coordination of expression of genes encoding enzymes falling in these categories. Also, it cannot be ruled out that organ specificity may be driven by specific candidate SP effectors, rather than specific enzymatic capacity. Such organ-specific effectors were indeed reported in other fungal plant pathogens [44].

In overwintering macrocyclic rust fungi, ultrastructural microscopy observations showed that meiosis is blocked in prophase I at the diplonema stage and restart after germination in spring, consistent with expression profiling of karyogamy and meiosis-related genes [11, 12]. Ultrastructural analysis of *Gymnosporangium* teliospore maturation and basidiospores formation showed that meiosis begins very shortly after karyogamy. Also, meiosis is not interrupted because teliospores of *Gymnosporangium* germinate without a period of dormancy [10]. Some early stage karyogamy-related genes reported in *M. larici-populina* teliospores are not detected in *G. japonicum*, neither in *G. asiaticum or G. yamadae* [22] which implies that this process already took place in collected teliospores. Meiotic genes conserved in eukaryotic species [45] are found in *G. japonicum* teliospores. Meiosis anaphase genes are highly expressed in *G. japonicum*, *G. asiaticum* and *G. yamadae*, but not detected in *M. larici-populina*. For instance, genes encoding the meiotic cell division protein Dom34 and the checkpoint serine/threonine-protein kinase Bub1 are expressed in *Gymnosporangium* teliospores. Bub1 regulates chromosome segregation [46] and Dom34 is responsible for cell division [45]. These observations likely illustrate the differences in the timing of karyogamy and meiosis processes ongoing in teliospores of macrocyclic and demicyclic rust fungi. Evidence from a larger number of species is now needed to determine to which extent the onset of these biological processes differs between rust fungi with different life cycles.

Recent phylogenetic analysis revealed that the *Gymnosporangium* group is not confamilial with other members of Pucciniaceae as previous thought and more likely represents a family-level lineage [5]. Phylogenomics comparison of three *Gymnosporangium* spp. transcriptomes with the genomes of three rust species selected in different taxonomical families of the Pucciniales using predicted proteomes shows that *Gymnosporangium* stands on an independent branch with more proximity to Pucciniaceae than any other rust families. This comparison also confirms that *G. japonicum* is closer to *G. yamadae* than *G. asiaticum*, consistent with previous phylogenetic findings [6, 47, 48]. This phylogenomic approach also reveals proteins that are specific at different taxonomical levels, although it is not possible to definitively conclude on the extent of protein families from the transcriptome that is only the expressed portion of the gene complement in a given species. The comparison to the genomes of *C. quercuum* f. sp. *fusiforme*, *M. larici-populina*, *P. graminis* f. sp. *tritici* [28–30] reveals the presence of 1514 protein clusters shared at the order level in the MCL analysis, of which 305 are specific to Pucciniales after comparison with the nr database. Most of these specific genes are of unknown function ([5]; this study), nevertheless, the detection of their expression at this particular stage indicates that they likely play a role in teliospores, which brings new information to better delineate biology of rust fungi. Information about expression in teliospores is still lacking for some of the selected rust genomes and future studies are needed to better describe the genetic program expressed at this particular spore stage. Also, differences observed between demicyclic and macrocyclic rust fungi for key cellular processes like karyogamy and meiosis ([11, 22], this study), show that temporal dissection of spore maturation process is needed in rust fungi. On the other hand, the phylogenomic comparison using the MCL approach helps to identify genes that are either specific across the *Gymnosporangium* genus or specific to each *Gymnosporangium* species. It is tempting to speculate that some of these genes may be related to the telial host that is common to the three species. Future comparison to *Gymnosporangium* species infecting other gymnosperms will be needed to test this hypothesis and validate host-specific genes. Among highlighted specific genes, SPs represent candidate effectors that may play a role in infection of the aecial host by basidiospores. Identification of effectors is important to support progress in resistance deployment in host plants [7], particularly in Chinese apple orchards where *G. yamadae* causes major damages [49]. Discrimination of SPs in each *Gymnosporangium* spp. helps to narrow down the list of specific candidates and will support future effector studies to better understand the biology of infection as demonstrated for other rust species [50–52]. The transcriptome of *G. japonicum* and its comparison to other *Gymnosporangium* species bring new valuable information to gain insights into the biology of this genus of rust fungi poorly explored at the molecular level, as well as for the biology of Pucciniales in general since it focuses on a spore stage that is so far overlooked. Rust genomes are large and highly repetitive, and can be of several hundred megabases in size [5]. For instance, the genome size of the close-relative species *Gymnosporangium confusum* has been estimated at 893 Mb [14]. The genome size of other *Gymnosporangium* species is not yet determined but might be substantially large and may require the use of single molecule sequencing technologies, such as PacBio or Oxford Nanopore, to achieve complete sequencing [5]. These transcriptomic surveys are only the first glimpse into *Gymnosporangium* genomics and the next stage will be the sequencing of a representative genome to support progress in understanding pathogenesis of these fungi.

## Conclusions

We presented the transcriptome analysis of *G. japonicum* teliospores collected from juniper tree and we conducted a comparative transcriptomic analysis with other *Gymnosporangium* spp. at the same stage of the life cycle. This stage is mostly overlooked in rust fungi and the complementary information provided here suggests that it is comparable across *Gymnosporangium* species in term of expressed cellular categories, likely to prepare for production of the next spore stage in the life cycle. Differences in the expression profiles of karyogamy and meiosis related genes in teliospores of *Gymnosporangium* species and in overwintering macrocyclic rust fungi likely highlight the different timing of related biological processes in these relative species. Comparison of *Gymnosporangium* transcriptomes to other Pucciniales fungi unravelled lineage-specific protein families, including *Gymnosporangium* spp. specific candidate secreted effectors expressed in teliospores, that remain to be functionally addressed.

## Methods

### Sample collection

Branch samples of juniper (*J. chinensis* L.) infected by *G. japonicum* were collected from the Yat-sen Mausoleum, Nanjing, Jiangsu, China, in March 2016. Telia are visible under the form of dark brown teliospore masses under the bark (Fig. 1). The identity of the rust fungus was confirmed by microscopy and by molecular identification (using ITS and TEF1; Tao S and Liang Y-M, unpublished observations). Batches of 50 mg of mature teliospores were recovered with a needle by gently scratching from beneath the bark of the branch. Three replicate samples were collected in sterile tubes. The samples were immediately snap frozen in liquid nitrogen and then stored at − 80 °C until processed.

### RNA-sequencing and de novo assembly

Total RNA was isolated from 50 mg mature teliospores by using TRIzol Reagent (Invitrogen, CA, USA) and was subjected to a DNase treatment with the DNA-free™ DNA Removal Kit (Ambion, Austin, TX, United States) according to the furnisher’s instructions. The teliospores were ground to powder with sterile mortar and pestle in liquid nitrogen. RNA was stored in DEPC-treated water. RNA quality and quantity were determined with a NanoPhotometer® spectrophotometer (IMPLEN, CA, USA) and a Qubit® RNA Assay Kit in Qubit® 2.0 Fluorometer (Life Technologies, CA, USA). RNA integrity (RIN) was assessed using the RNA Nano 6000 Assay Kit of the Agilent Bioanalyzer 2100 system (Agilent Technologies, CA, USA). Three cDNA libraries of *G. japonicum* (DNJGJ_1, DNJGJ_2 and DNJGJ_3) were constructed with specific 6-bp nucleotide bar-coding tags, using a NEBNext® Ultra™ RNA Library Prep Kit for Illumina (NEB, USA) following the manufacturer’s protocols. Tagged cDNA libraries were pooled in equal ratios and used for 100-bp PE sequencing on the Illumina HiSeq2000 platform (Illumina, San Diego, CA, USA) at Novogene Bioinformatics Technology Co., Ltd. (Beijing, China). Raw reads from fastq files were firstly processed through in-house perl scripts at Novogene Bioinformatics Technology Co. In this step, clean reads were obtained by removing adapters, poly-N, and low-quality reads (based on Q20, Q30, GC-content and sequence duplication level using the Trimmomatic software [53];) from the raw data. The left files (read1 files) from all three libraries were pooled into one big left.fq file, and right files (read2 files) were pooled into one big right.fq file. A reference transcriptome assembly was determined based on the left.fq and right.fq using Trinity [23] with min_kmer_cov set to 2 by default and all other parameters set to default. Following the construction of the reference transcriptome, clean data from individual replicates were mapped back onto the assembled transcriptome (unigenes) and then read count for each gene was obtained from the mapping results, and gene expression levels were estimated by the software package RSEM, with Bowtie2 as a read aligner (default parameters, mismatch: 0) [24] for each library. Transcriptome assembly; unigene determination and putative CDS prediction; read count levels for the three replicates; and proteome prediction from unigenes were performed at Novogene Bioinformatics Technology Co following procedures previously described, using the same bioinformatic programs and versions [22].

### Gene functional annotation

In order to determine the functional categories of unigenes, homology searches by BLASTX were performed against public databases (NCBI blast+ 2.2.28; cut-off e-value<1e-5), including NCBI nr and nt, KOG, KEGG pathways orthology and, Swissprot and Pfam databases. During assignment to KOG category, we added a KOG category termed “Secreted proteins” that consists of secreted proteins of unknown function as described in [16].

### Prediction of secreted proteins

The secretome of *G. japonicum* was determined from the proteome using a custom pipeline combining different bioinformatic programs, including SignalP v.4, WolfPSort,Tmhmm, TargetP and PS-Scan algorithms as reported by [26]. The presence of nuclear localization signal (NLS) was defined with PredictNLS [54]. In order to accurately compare predicted secretomes from *G. japonicum* (this study) and from *G. yamadae* and *G. asiaticum* (previously published by [22]), the same pipeline was strictly applied to the three species. The predicted proteomes derived from the unigenes were used to search putative CAZymes in the dbCAN v2.0 HMM-based CAZy annotation server [55]. For the three predicted secretomes, functional annotations were also performed with Merops [56] and the Lipase Engineering database [57].

### Gene family construction and Phylogenomics analysis

The complete proteomes of four fungi belonging to the Pucciniomycotina with available reference genome sequences were downloaded from the Joint Genome Institute Mycocosm website (https://genome.jgi.doe.gov/mycocosm/home; [58]): *Cronartium quercuum* f. sp. *fusiforme* G11 version 1.0 [30], *Melampsora larici-populina* v2 [28], *Puccinia graminis* f. sp. *tritici* CEL 75–36–700-3 race SCCL v2 [29]. *Microbotryum lychnidis-dioicae* p1A1 Lamole [31]. The three proteomes predicted for *Gymnosporangium* species (*G. japonicum*, this study, and *G. yamadae* and *G. asiaticum* from [22]) were used for comparison with reference rust proteomes. Gene families were clustered with fastOrtho MCL v12.135 [34] using inflation parameters of 3 and 50% identity and coverage.

The program Benchmarking Universal Single-Copy Orthologs (BUSCO, [32]) was independently run for the three *Gymnosporangium* transcriptome assemblies and the three selected Pucciniales genomes and in *M. lychnidis-dioicae* (Microbotryales), in order to detect the single copy genes from each assembly. The amino acid sequence of families found in all species were aligned using MAFFT and the alignments was then used to construct a phylogenomic tree using RAxML with 1000 bootstraps and the PROTGAM-MAAUTO substitution model [33]. The resulting tree was rooted with *M. lychnidis-dioicae* and visualized in Geneious and labels were manually placed to improve legibility.

## Supplementary information

**Additional file 1: Figure S1.** General read sequences and mapping information (top table); transcripts, unigenes and full-length CDS description (bottom table); and unigene length distribution (bottom graph) obtained for the *G. japonicum* teliospores transcriptome. The same information for the species *G. asiaticum* and *G. yamadae* were extracted from [22] and added side to side with *G. japonicum* to allow for direct metrics comparison. (PDF 345 kb)

**Additional file 2: Supplementary Data 1.** Fasta file containing 40,583 *G. japonicum* transcript sequences. (FASTA 45632 kb)

**Additional file 3: Supplementary Data 2.** Fasta file containing 30,243 *G. japonicum* unigene sequences. (FASTA 24636 kb)

**Additional file 4: Table S1.** FPKM values and annotation results for each assembled *G. japonicum* unigene. (XLSX 7706 kb)

**Additional file 5: Figure S2.** Density distribution profile of FPKM values in the three *G. japonicum* replicate libraries (DNJGJ_1, DNJGJ_2 and DNJGJ_3). (PDF 70 kb)

**Additional file 6: Table S2.** KOG and KEGG annotation and classification of *G. japonicum* unigenes. (XLSX 93 kb)

**Additional file 7: Figure S3.** Distribution of FPKM values for *G. japonicum* unigenes. The numbers of unigenes more expressed than each Log_10_(FPKM) level are detailed. Log_10_(FPKM) are used as arbitrary separations of highly, moderately and less expressed gene categories. (PDF 5281 kb)

**Additional file 8: Table S3.** CAZymes annotation results for *Gymnosporangium* unigenes in the dbCAN v2.0. AA, CAZyme with auxiliary activities; CBM, carbohydrate binding module; CE, carbohydrate esterase; GH, glycosyl hydrolase; GT, glycosyl transferase; PL, polysaccharide lyase. FPKM and Log_10_FPKM values are reported along CAZyme annotation. (XLSX 42 kb)

**Additional file 9: Figure S4.** Distribution of *G. japonicum*, *G. yamadae* and *G. asiaticum* predicted proteins in CAZymes families in dbCAN v2.0. (PDF 27 kb)

**Additional file 10: Table S4.** Reciprocal best Blastp hits results between *Gymnosporangium* spp. predicted proteomes. GYA, *G. asiaticum*; GYJ, *G. japonicum*; GYY, *G. yamadae*. (XLSX 627 kb)

**Additional file 11: Table S5.** Secreted proteins predicted in *G. japonicum*, *G. yamadae* and *G. asiaticum*. CPL stands for CAZymes, proteases and lipases homology prediction; SP is for swissprot blastp homology search; perc C for the percentage of cysteine residues in the amino acid sequence and NLS stands for Nuclear Localisation Signal prediction. (XLSX 261 kb)

**Additional file 12: Table S6.** *Gymnosporangium* spp. specific clusters identified by Markov Cluster Algorithm (MCL). Cluster IDs are provided and secretion prediction is indicated. Gymas/GYA, *G. asiaticum*; Gymja/GYJ, *G. japonicum*; Gymya/GYY, *G. yamadae*. (XLSX 276 kb)

**Additional file 13: Table S7.** Clusters of secreted proteins for the three *Gymnosporangium* species. Cluster ID from the MCL analysis; prot nb, number of proteins in the given cluster; SP nb, number of predicted secreted proteins in the cluster; and detailed protein ID for secreted proteins. Novel secreted proteins with no hit in the NCBI nr database are indicated. (XLSX 13 kb)

## Data Availability

All raw sequence data generated in this study is deposited in the NCBI Sequence Read Archive (https://submit.ncbi.nlm.nih.gov/subs/sra/) under the accession number SRR8906254-SRR8906256. Additional previously published transcriptome data analyzed during this study are included in [22] and its supplementary information files. All other supporting data are included as additional files.

## Abbreviations

CAZymes: Carbohydrate active enzymes
FPKM: Fragment per kilobase per million mapped reads
GHs: Glycosyl hydrolases
GO: Gene ontology
KEGG: Kyoto encyclopaedia of genes and genomes
KOG: Eukaryotic orthologous groups
MCL: Markov Cluster Algorithm
nr: NCBI non-redundant database
nt: NCBI Nucleotide database
Pfam: Protein family
RSEM: RNA-Seq by Expectation Maximization
RTP1: Rust Transferred Protein 1
SPs: Secreted proteins
SSPs: Small secreted proteins
