# Supplementary material for: Comparative transcriptomics of Gymnosporangium spp. teliospores reveals a conserved genetic program at this specific stage of the rust fungal life cycle
Source: BMC Genomics. 2019 Oct 9;20:723. doi: 10.1186/s12864-019-6099-x (PMC6785864; doi:10.1186/s12864-019-6099-x)
Supplement: Supplementary file 1 — Additional file 1: Figure S1. General read sequences and mapping information (top table); transcripts, unigenes and full-length CDS description (bottom table); and unigene length distribution (bottom graph) obtained for the G. japonicum teliospores transcriptome. The same information for the species G. asiaticum and G. yamadae were extracted from [22] and added side to side with G. japonicum to allow for direct metrics comparison. (PDF 345 kb) [file 12864_2019_6099_MOESM1_ESM.pdf]

Species	Sample	Raw Reads	Clean Reads	Clean Bases	GC Content (%)	Mapping read (rate %)
G. japonicum	DNJGJ_1	49,245,136	48,665,186	7.30G	49.72	40,327,824 (82.87%)
	DNJGJ_2	60,133,718	59,507,808	8.93G	50.24	50,470,520 (84.82%)
	DNJGJ_3	52,751,538	52,175,576	7.83G	50.35	44,211,226 (84.75%)
G. yamadae	DSXGY_1	47,629,874	46,917,624	7.04G	48.71	38,545,026 (82.29%)
	DSXGY_2	59,428,164	58,748,388	8.81G	51.40	50,102,470 (85.29%)
	DSXGY_3	49,485,212	48,752,948	7.31G	48.41	41,058,330 (84.35%)
G. asiaticum	DSXGA_1	45,287,548	44,616,412	6.69G	53.63	33,171,198 (74.36%)
	DSXGA_2	52,290,920	51,638,726	7.75G	52.58	39,759,730 (77.01%)
	DSXGA_3	66,334,424	65,471,582	9.82G	53.84	54,721,096 (83.59%)

Statistics of RNA-seq reads for each library and mapping of reads using three reference transcriptomes

	G. japonicum	*G. yamadae*	*G. asiaticum*
Transcripts	40,583	49,318	54,742
Max length	15,436	19,126	16,773
Average length	1,059	1,006	1,059
N50	2,079	1,957	2,070
Total residues	42,991,958	49,601,912	57,968,265
Unigenes	30,243	35,102	36,343
Max length	15,436	19,126	16,773
Average length	809	756	754
N50	1,756	1,654	1,664
Total residues	24,460,547	26,521,164	27,406,256
Full-length CDS	15,722	18,467	17,769
Mean length	795	848	724
>90 bp	15,653 (99.6%)	18,303 (99.1%)	17,685 (99.5%)

Summary of transcriptome data for three *Gymnosporangium* teliospores

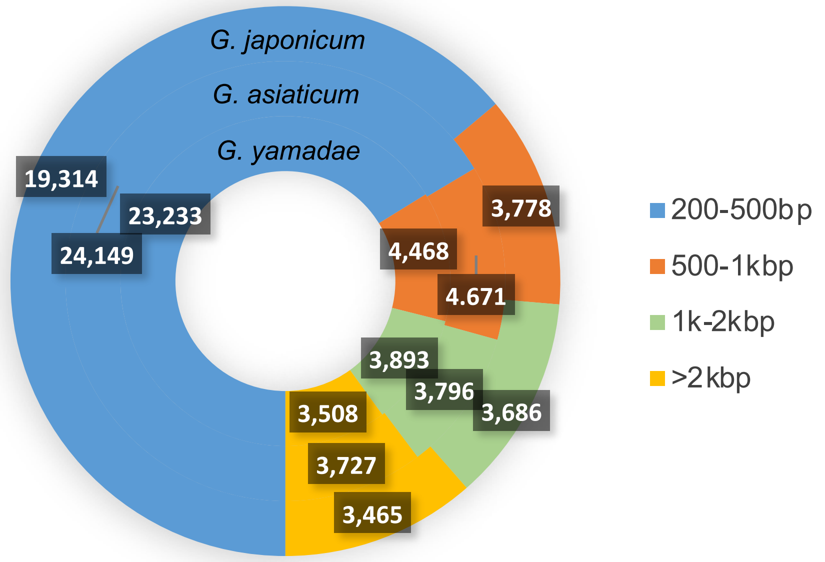

Unigene length distribution
