# Supplementary figures and images for: Comparative transcriptomics of Gymnosporangium spp. teliospores reveals a conserved genetic program at this specific stage of the rust fungal life cycle
Source: BMC Genomics. 2019 Oct 9;20:723. doi: 10.1186/s12864-019-6099-x (PMC6785864; doi:10.1186/s12864-019-6099-x)

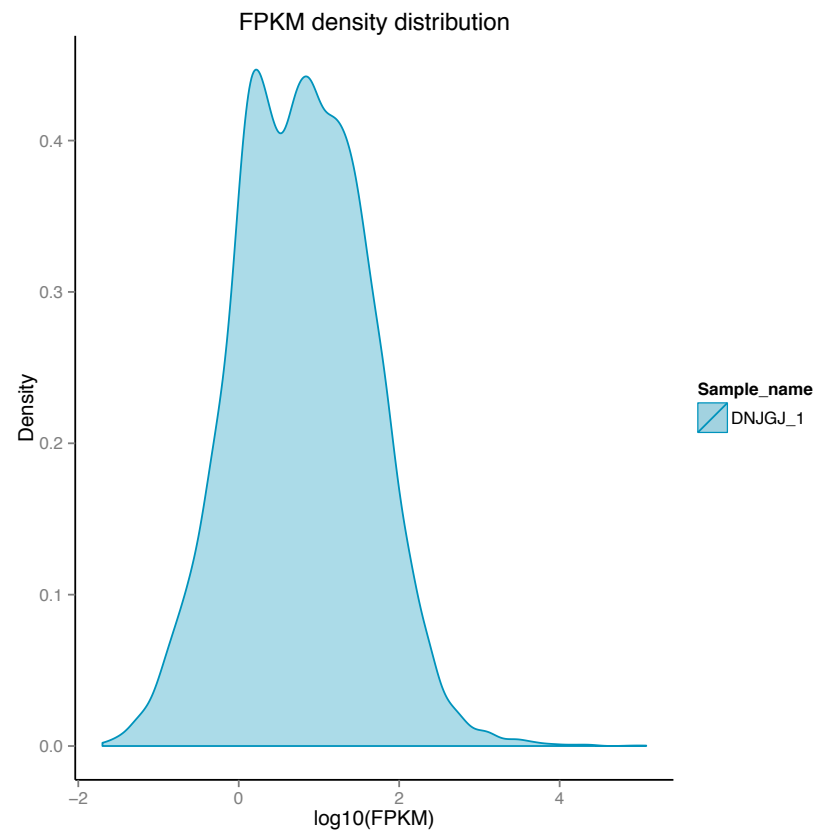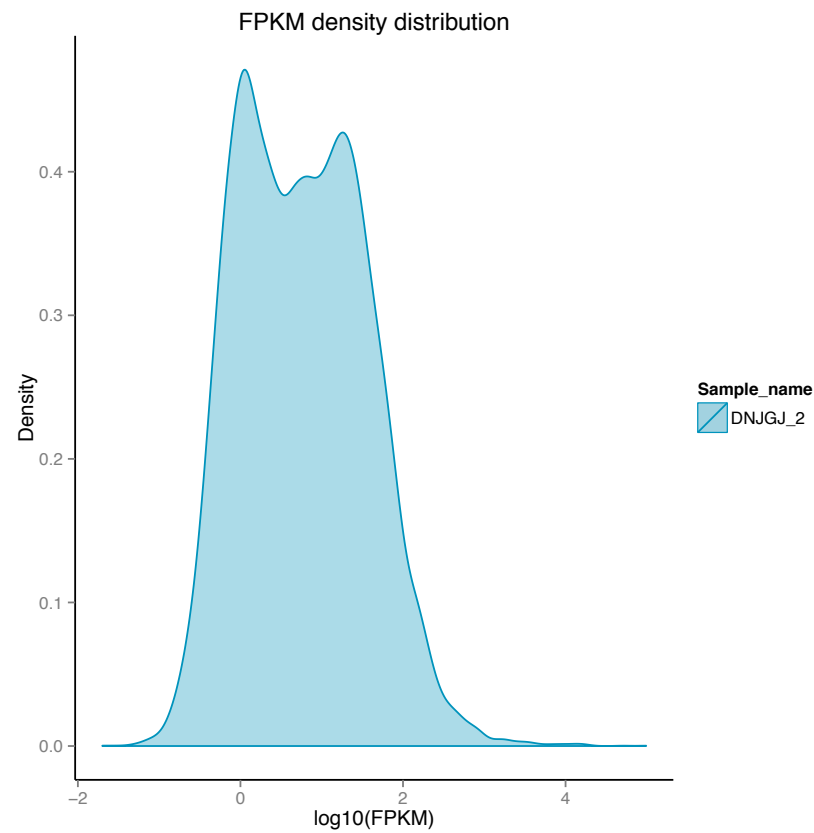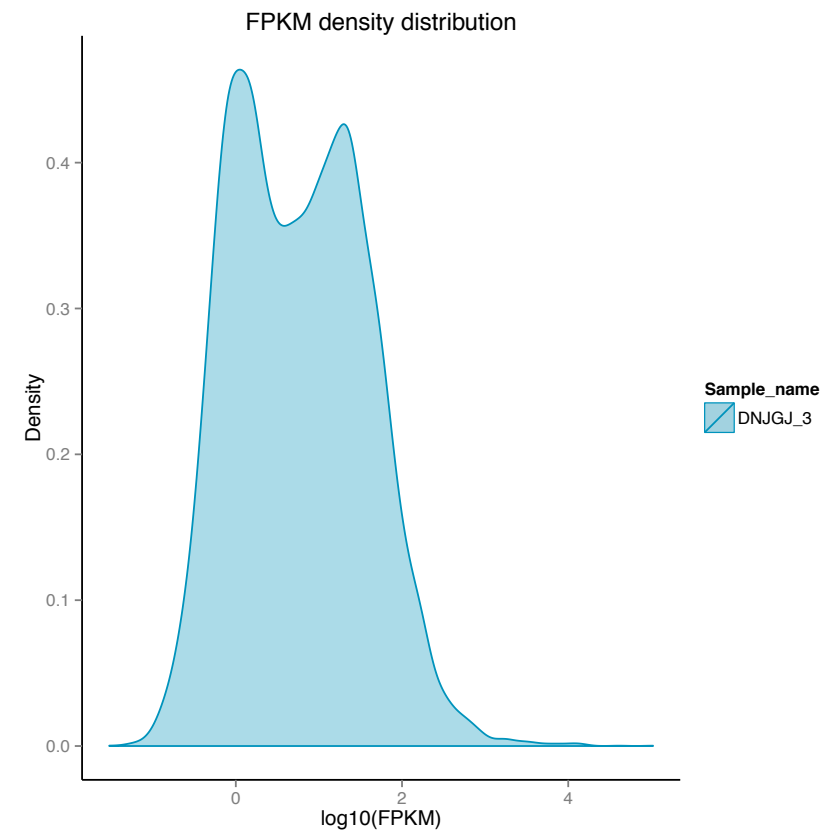

Supplement: Supplementary file 5 — Additional file 5: Figure S2. Density distribution profile of FPKM values in the three G. japonicum replicate libraries (DNJGJ_1, DNJGJ_2 and DNJGJ_3). (PDF 70 kb) [file 12864_2019_6099_MOESM5_ESM.pdf]

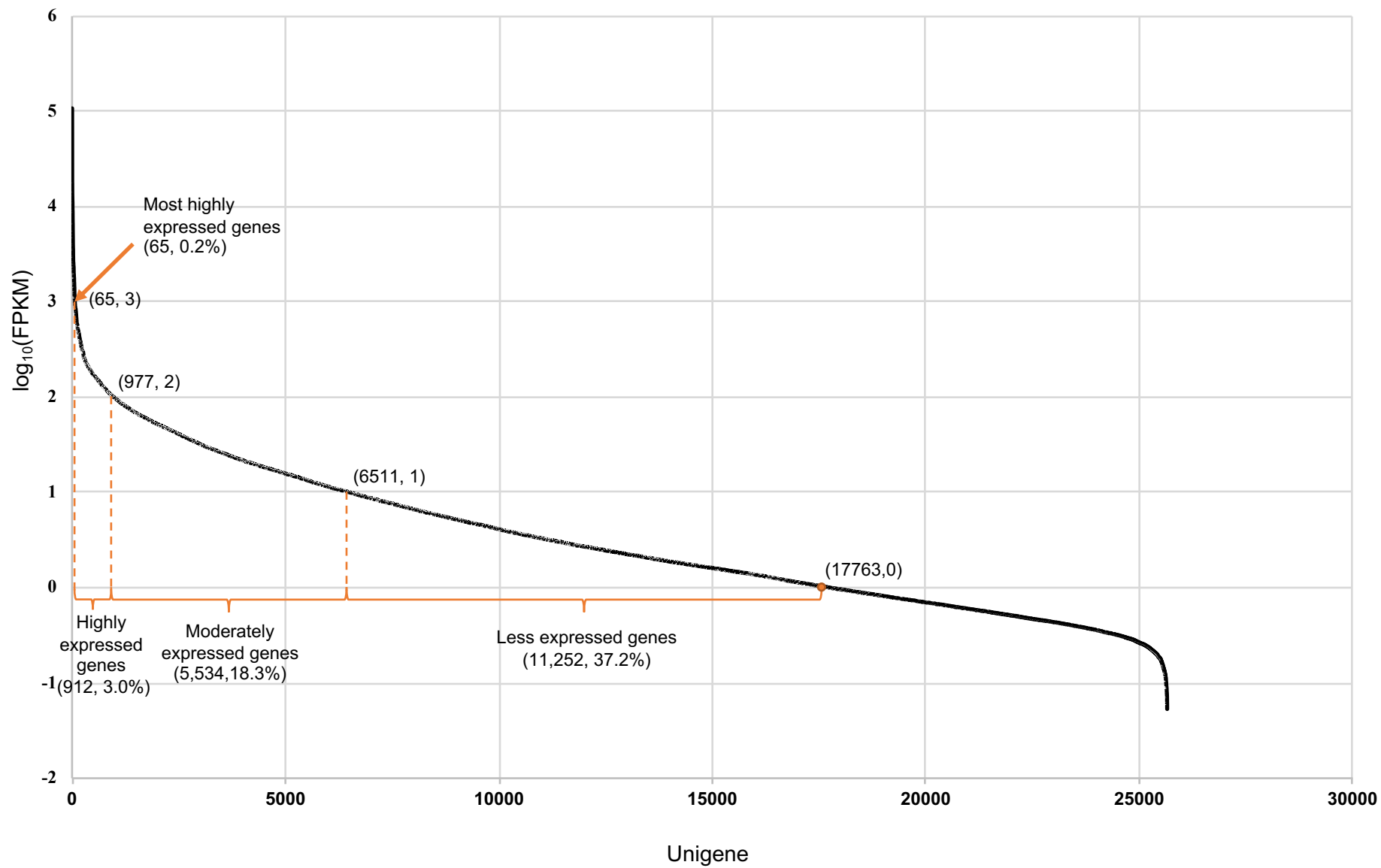

Supplement: Supplementary file 7 — Additional file 7: Figure S3. Distribution of FPKM values for G. japonicum unigenes. The numbers of unigenes more expressed than each Log10(FPKM) level are detailed. Log10(FPKM) are used as arbitrary separations of highly, moderately and less expressed gene categories. (PDF 5281 kb) [file 12864_2019_6099_MOESM7_ESM.pdf]

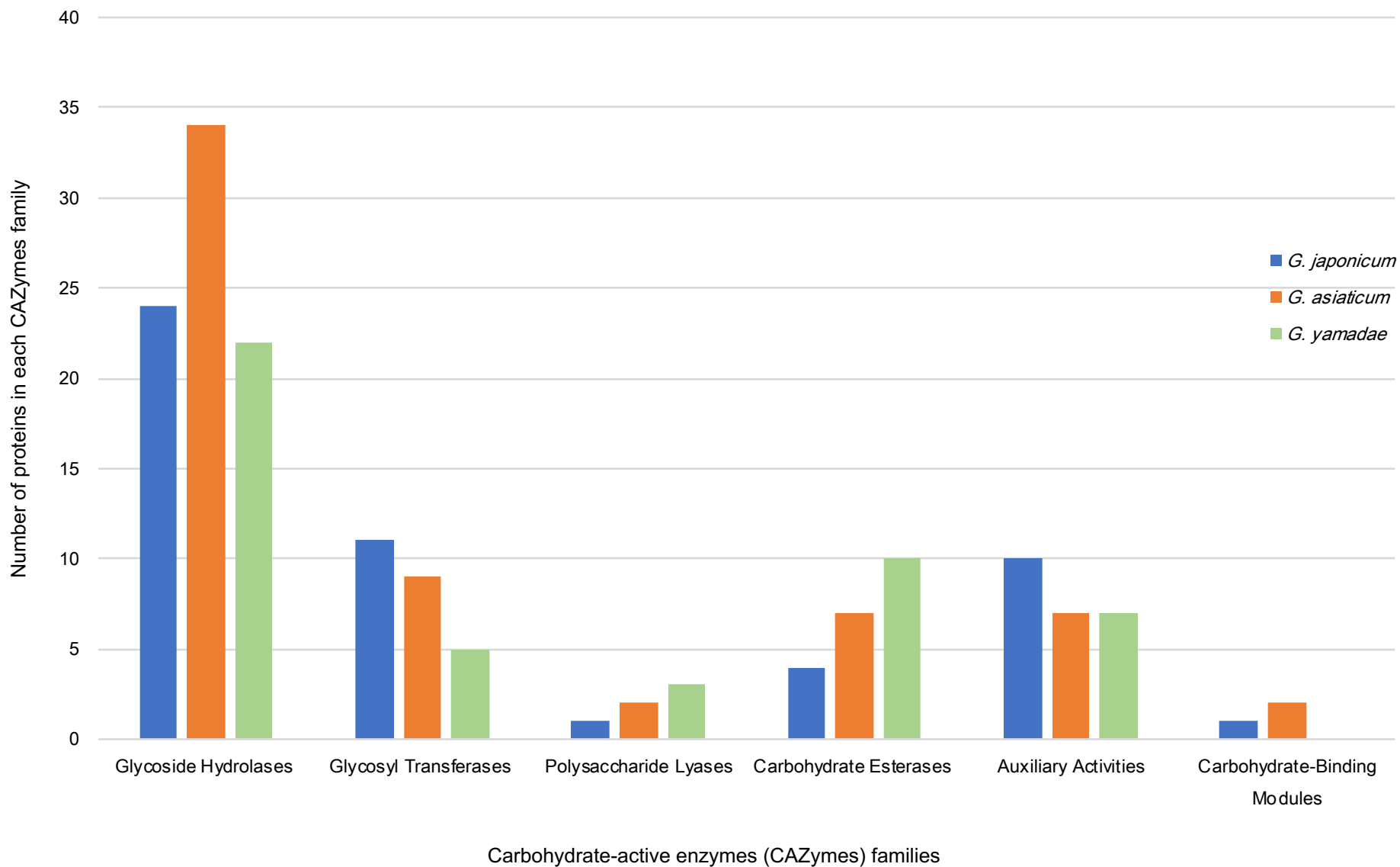

Supplement: Supplementary file 9 — Additional file 9: Figure S4. Distribution of G. japonicum, G. yamadae and G. asiaticum predicted proteins in CAZymes families in dbCAN v2.0. (PDF 27 kb) [file 12864_2019_6099_MOESM9_ESM.pdf]
